# Nonalcoholic Beverage Consumption and the Risk of Depression, Anxiety, and Stress: A Systematic Review and Meta‐Analysis

**DOI:** 10.1155/da/7404084

**Published:** 2026-08-31

**Authors:** Yixin Ouyang, Jingyi Liang, Xijie He, Xiangrui Song, Ting Qi, Mengkun Li, Yuan Feng, Songyu Wu, Qianlu Ding, Zhouyang Sun, Tingyi Jia, Changgui Kou, Wei Bai

**Affiliations:** ^1^ Department of Epidemiology and Biostatistics, School of Public Health, Jilin University, 1163 Xinmin Street, Changchun, Jilin Province, 130021, China, jlu.edu.cn

**Keywords:** anxiety, depression, meta-analysis, nonalcoholic beverages, stress

## Abstract

**Background:**

Depression, anxiety, and stress represent a major global public health burden. This systematic review and meta‐analysis evaluated the association between nonalcoholic beverage consumption and these mental health outcomes.

**Methods:**

We systematically searched four databases (PubMed, Web of Science, PsycINFO, and EMBASE) through November 19, 2024. Random‐effects models were used to calculate pooled odds ratios (ORs) with 95% confidence intervals (CIs), with subgroup analyses and meta‐regression to explore heterogeneity sources.

**Results:**

The analysis included 59 studies comprising 132 independent reports with 2,050,716 participants. Higher nonalcoholic beverage consumption was significantly associated with depression (OR = 1.18, 95% CI: 1.11–1.26) and anxiety (OR = 1.31, 95% CI: 1.12–1.55). For stress, the main pooled analysis was not statistically significant (OR = 1.15, 95% CI: 0.98–1.35), whereas sensitivity analysis excluding Sangsefidi et al. was significant (OR = 1.21, 95% CI: 1.06–1.38). Beverage‐specific analyses revealed that coffee was inversely associated with depression (OR = 0.87, 95% CI: 0.76–0.99), while sugar‐sweetened beverages, bubble tea, carbonated beverages, and energy drinks were positively associated with depression, and bubble tea and carbonated beverages were positively associated with anxiety.

**Conclusions:**

Nonalcoholic beverage consumption was associated with depression and anxiety, whereas evidence for stress was less stable. Coffee showed an inverse association with depression, while most other beverage types showed positive associations with depression or anxiety. These findings may help inform future mental health‐related dietary research and public health strategies, particularly regarding the assessment of beverage consumption patterns, while further prospective and interventional studies are needed before specific dietary recommendations can be made.

## 1. Background

Depression, anxiety, and stress represent a substantial global public health burden. According to World Health Organization reports, ~322 million people worldwide are affected by depressive disorders, while 264 million suffer from anxiety disorders, with the prevalence of stress‐related symptoms showing an increasing trend across all age groups [1, 2]. These mental health disorders not only impair individual functioning and quality of life but also impose significant socioeconomic costs.

The consumption of nonalcoholic beverages has emerged as a focus of research in exploring modifiable risk factors for depression, anxiety, and stress. As essential components of the daily diet, nonalcoholic beverages have demonstrated consistently increasing global consumption patterns. Evidence indicates that American adults consume over 1500 mL of beverages daily, predominantly consisting of coffee, tea, and sugar‐sweetened beverages [3]. This trend is reflected globally, with sugar‐sweetened beverage consumption increasing by 27% over recent decades [4], alongside substantial expansion of the energy drink market and annual coffee consumption exceeding 10 billion kilograms (https://ico.org/what-we-do/world-coffee-statistics-database/).

Current evidence regarding the relationship between nonalcoholic beverages and mental health reveals considerable inconsistencies. For coffee consumption, some studies have reported inverse associations with depression risk [5] and protective effects against anxiety [6], whereas others have found no significant relationships [7]. Research on sugar‐sweetened beverages yields similarly conflicting results, with studies reporting positive associations with depression risk [8, 9], while others detected no significant association [10]. The evidence concerning tea consumption presents an even greater complexity. Traditional teas such as green tea have shown protective effects in some studies [11], whereas emerging categories like bubble tea have demonstrated positive associations with anxiety symptoms [12, 13]. Energy and carbonated beverages similarly exhibit inconsistent patterns, with notable variations between studies by Pengpid and Peltzer [14] and Azagba et al. [15]. Particularly noteworthy are the differential susceptibilities observed across population subgroups. Adolescent populations appear more vulnerable to the effects of beverage consumption, as evidenced by several studies [16, 17].

Previous meta‐analyses in this field have been limited in scope. While several reviews have explored the relationship between beverage consumption and depression, no previous meta‐analysis has specifically and separately synthesized the evidence for anxiety or stress. Furthermore, existing syntheses have often been restricted to single beverage categories (e.g., sugar‐sweetened beverages or tea) [18, 19] and have either focused solely on depressive symptoms [20] or failed to distinguish between clinically diagnosed depression and depressive symptoms [21, 22]. Additionally, most previous meta‐analyses failed to conduct subgroup analyses by geographic region, limiting the understanding of potential regional variations in these associations.

Based on the above, we conducted this systematic review and meta‐analysis to comprehensively evaluate the associations between nonalcoholic beverage consumption and depression, anxiety, and stress, to separately analyze clinically diagnosed depression and depressive symptoms, and to explore the influence of sociodemographic factors and study characteristics on these associations. The findings will provide important evidence for developing targeted public health and clinical strategies.

## 2. Methods

### 2.1. Literature Search and Selection Criteria

This meta‐analysis was conducted in accordance with the Preferred Reporting Items for Systematic Reviews and Meta‐Analyses (PRISMA) [23] checklist and the Meta‐analysis of Observational Studies in Epidemiology (MOOSE) [24] guidelines (Supporting Information Table S1). The study protocol was registered in the International Platform of Registered Systematic Review and Meta‐analysis Protocols (INPLASY, https://inplasy.com/) with the Registration Number INPLASY2025110060. The protocol was registered after the initial database search and screening had commenced but before completion of the final analyses and manuscript revision; no deviations from the registered protocol occurred after registration.

We conducted a systematic search of four electronic databases (PubMed, Web of Science, PsycINFO, and EMBASE) from inception to November 19, 2024, to identify studies examining the association between nonalcoholic beverage consumption and depression, anxiety, and stress. The primary search terms were based on “nonalcoholic beverages,” “depression,” “anxiety,” and “stress.” For “nonalcoholic beverages,” we used the following terms: “coffee,” “tea,” “green tea,” “black tea,” “beverages,” “beverage,” “soft drink,” and “carbonated beverages.” For “depression,” we utilized terms such as “depress ^∗^,” “depression,” “depressive,” “depressed,” “depressive symptom,” “emotional depression,” “depression disorder,” and “depression risk.” For “anxiety,” we employed the following search terms: “anxiety,” “anxiety ^∗^,” and “anxieties.” The search term for “stress” was “stress ^∗^.” The detailed search strategy is provided in Supporting Information Table S2.

After removing duplicates, two researchers (Yixin Ouyang and Jingyi Liang) independently screened the title and abstracts to exclude irrelevant publications, such as reviews, conference papers, letters, book chapters, and literature unrelated to the research topic. The remaining full‐text articles were assessed for eligibility based on predefined inclusion and exclusion criteria. Any discrepancies during the screening process were resolved through discussion or by a third researcher (Wei Bai).

In accordance with the PECOS acronym, studies included must meet the following criteria: Population (P): general population (adults and/or adolescents). Exposure (E): nonalcoholic beverage consumption assessed in ≥2 quantitative categories. Comparator (C): the lowest consumption category or nonconsumers. Outcome (O): depression, anxiety, or stress symptoms/diagnosis determined through validated scales, clinical interviews, or ICD/DSM codes; reporting odds ratios (ORs), risk ratios (RRs), or hazard ratios (HRs) with 95% confidence intervals (CIs). Study design (S): cross‐sectional or prospective cohort studies.

The exclusion criteria included (1) studies not reporting effect sizes or where effect sizes could not be converted to OR/RR/HR; (2) studies examining mixed alcoholic and nonalcoholic beverages without separable data; (3) duplicate database records from the same cohort wave; (4) studies focusing on the relationship between tea consumption and depression, as the causal association in this regard has been established by Mendelian randomization evidence; and (5) studies focusing exclusively on pregnant or lactating women.

### 2.2. Data Extraction and Quality Assessment

Comprehensive information was extracted from the included studies, encompassing the following information: first author, publication year, title, journal name, region, country, study period, study design, sampling method, sample size, proportion of male participants, outcome measures, outcome assessment methods, and effect estimates with corresponding 95% CIs. Two researchers (Yixin Ouyang and Jingyi Liang) independently conducted data extraction with cross‐verification to ensure consistency. Any discrepancies were resolved by a third researcher (Wei Bai).

When both unadjusted and adjusted ORs were provided in a study, priority was given to the adjusted estimates to assess the association between nonalcoholic beverages and depression, anxiety, and stress. Studies that did not directly report ORs but provided convertible effect measures (e.g., raw 2 × 2 tables or other statistics from which ORs could be directly calculated) were included. Conversely, studies reporting only per‐unit increment linear regression coefficients that could not be converted to relative risk estimates were excluded. When multiple exposure cut‐offs were used, we extracted the most extreme contrast (highest vs. lowest category) to maximize comparability across studies. Dichotomous outcomes were directly utilized, whereas multicategory exposure studies were analyzed using statistics comparing extreme intake groups. When multiple reports shared the same study population, the most comprehensive study was selected for inclusion. To address potential multiplicities in reporting, individual studies with multiple exposures or outcomes were treated as separate entries in the meta‐analysis.

For cross‐sectional studies, we utilized the 11‐item checklist recommended by the Agency for Healthcare Research and Quality (AHRQ) to evaluate the methodological quality of the included studies [25, 26]. Each item was scored “0” for responses of “NO” or “UNCLEAR” and “1” for “YES.” The quality assessment criteria were as follows: low quality = 0–3 points; moderate quality = 4–7 points; and high quality = 8–11 points.

Regarding cohort studies, we employed the Newcastle–Ottawa Scale (NOS) to assess the quality of the included studies [25, 26]. The NOS comprises three domains with a total of nine items: selection (0–4 points), comparability (0–2 points), and exposure (0–3 points). In the “selection” and “exposure” categories, each item could earn up to one star, while the “comparability” category could earn up to two stars. Based on the total scores, studies were classified into three quality categories: 0–3 as low quality, 4–6 as moderate quality, and 7–9 as high quality.

### 2.3. Statistical Analysis

We extracted adjusted ORs from the original studies. RRs and HRs reported in previous studies were directly treated as equivalent to ORs for consistency [27]. Fixed‐effect or random‐effect models were employed to calculate pooled ORs and 95% CIs for the associations between nonalcoholic beverages and depression, anxiety, and stress. Heterogeneity was assessed using the *I* ^2^ statistic. A random‐effects model was employed when *I* ^2^ exceeded 50%, indicating substantial heterogeneity; otherwise, a fixed‐effect model was used [28].

To explore potential sources of heterogeneity, both subgroup analyses and meta‐regression were conducted. Subgroup analyses were conducted based on the following categorical variables: the definition of depression (clinically diagnosed depression as determined by a clinician vs. depressive symptoms assessed using rating scales), income levels and geographical regions from the World Bank (https://data.worldbank.org/country), sample size (dichotomized using a median split) [29], age distribution, COVID‐19 period, and type of nonalcoholic beverage. Additionally, sex‐specific estimates reported separately in the original studies were extracted and meta‐analyzed independently to elucidate potential sex differences. Meta‐regression analyses were conducted for continuous variables, including the proportion of male participants and the study quality score.

Sensitivity analysis was performed by examining the reliability of results after excluding each study one by one to assess the stability of the associations between nonalcoholic beverage intake and depression, anxiety, and stress [30]. Publication bias was assessed visually using funnel plots and statistically using Egger’s test [23, 31]. All statistical analyses in this study were performed using R software (Version 4.3.3) with the “meta” and “forestplot” packages, with a two‐tailed p‐value < 0.05 considered statistically significant for all tests.

## 3. Results

### 3.1. Search Results and Study Characteristics

**Figure 1:**
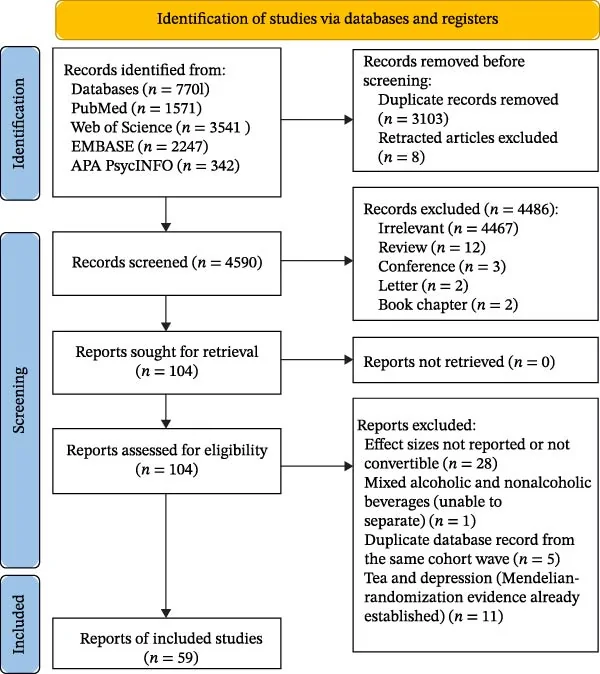
Flowchart of study selection procedure.

The systematic search identified 7701 potentially relevant records from four electronic databases. After duplicate removal and multistage screening of titles, abstracts, and full texts according to the established inclusion and exclusion criteria, 59 studies comprising 132 independent reports were included, encompassing a total of 2,050,716 participants (Figure 1). The included studies encompassed 22 countries across multiple continents, with the corresponding OR values for nonalcoholic beverage consumption and mental health (depression, anxiety, or stress) shown in Supporting Information Figure S1. Quality assessment categorized 12 studies (20%) as high quality, 43 studies (71.67%) as moderate quality, and 5 studies (8.33%) as low quality (Supporting Information Tables S4 and S5). Detailed characteristics of the included studies are presented in Supporting Information Table S3.

### 3.2. Nonalcoholic Beverages Consumption and Risk of Depression

Meta‐analysis of 59 studies (92 reports) revealed that nonalcoholic beverage consumption was associated with higher odds of depression (OR [95% CI]: 1.18 [1.11–1.26], *p* < 0.001), with considerable heterogeneity observed across studies (*I* ^2^ = 93.7%) (Supporting Information Figure S2). Stratification by study design indicated that cross‐sectional studies supported this positive association (OR [95% CI]: 1.20 [1.12–1.30], *p* < 0.001), whereas the association in cohort studies was not statistically significant (RR [95% CI]: 1.09 [0.98–1.23], *p* < 0.001) (Table 1).

**Table 1:** Summary of associations between nonalcoholic beverage consumption and risk of depression, anxiety, and stress.

Outcome	Subgroups	OR/RR (95% CI)	No. of reports	*p*‐Value	*I* ^2^ (%)
Depression	Cross‐sectional study	1.20 (1.12–1.30)	74	<0.001	92.2
	Cohort study	1.09 (0.98–1.23)	18	<0.001	86.5
Anxiety	Cross‐sectional study	1.31 (1.10–1.55)	28	<0.001	92.0
	Cohort study	1.48 (1.10–1.55)	1	—	—
Stress	Cross‐sectional study	1.15 (0.98–1.35)	11	<0.001	96.5
	Cohort study	—	—	—	—

Subgroup analysis by depression definition indicated that the association was consistent for both clinically diagnosed depression (OR [95% CI]: 1.12 [1.03–1.23], *p* < 0.001) and depressive symptoms (OR [95% CI]: 1.23 [1.14–1.34], *p* < 0.001), with the latter showing a slightly higher effect size. Subgroup analyses demonstrated that studies with sample sizes larger than 5,000 maintained a positive association (OR [95% CI]: 1.23 [1.14–1.32], *p* < 0.001). Both males (OR [95% CI]: 1.24 [1.12–1.38], *p* < 0.001) and females (OR [95% CI]:1.14 [1.03–1.27], *p* < 0.001) showed statistically significant positive associations (Supporting Information Figure S3).

**Figure 2:**
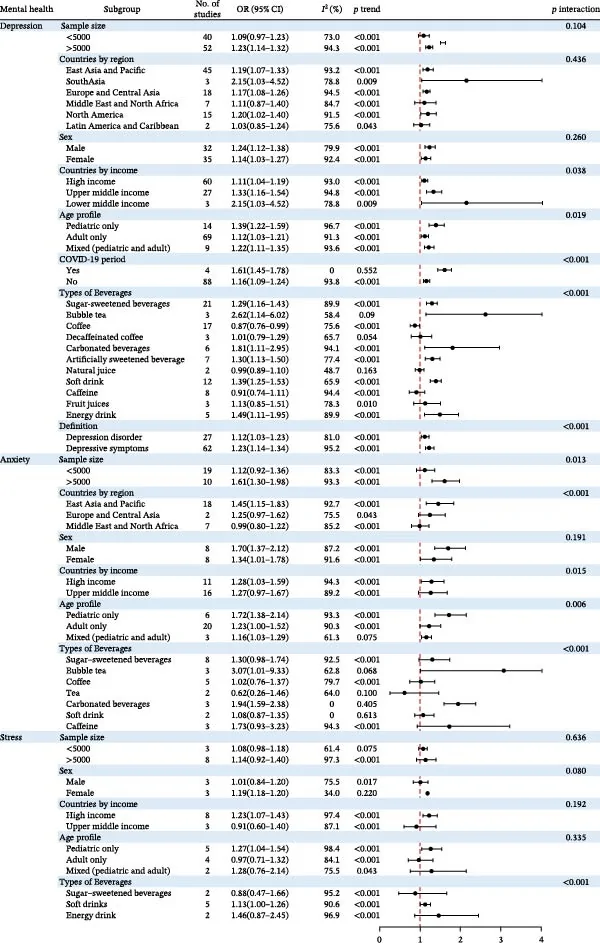
Forest plot of subgroup analyses of the association between nonalcoholic beverages and depression, anxiety, and stress.

Geographically, studies conducted in East Asia and Pacific, South Asia, Europe and Central Asia, and North America reported significant positive associations, with the strongest effect observed in South Asia (OR [95% CI]: 2.15 [1.03–4.52]). Higher association estimates were also identified in lower‐middle‐income countries (OR [95% CI]: 2.15 [1.03–4.52], *p* = 0.009) and pediatric‐only populations (OR [95% CI]: 1.39 [1.22–1.59], *p* < 0.001). Notably, studies conducted during the COVID‐19 pandemic reported a higher point estimate (OR [95% CI]: 1.61 [1.45–1.78]), although this result did not reach statistical significance when compared across subgroups. Beverage‐type subgroup analysis indicated that sugar‐sweetened beverages (OR = 1.29, 95% CI: 1.16–1.43), bubble tea (OR = 2.62, 95% CI: 1.14–6.02), carbonated beverages (OR = 1.81, 95% CI: 1.11–2.95), soft drinks (OR = 1.39, 95% CI: 1.25–1.53), artificially sweetened beverages (OR = 1.30, 95% CI:1.13–1.50), and energy drinks (OR = 1.30, 95% CI:1.13–1.50) were positively associated with depression risk, whereas coffee exhibited an inverse association (OR [95% CI]: 0.87 [0.76–0.99], *p* < 0.001). Differences across beverage subtypes were statistically significant (*p* < 0.001) (Figure 2).

**Figure 3:**
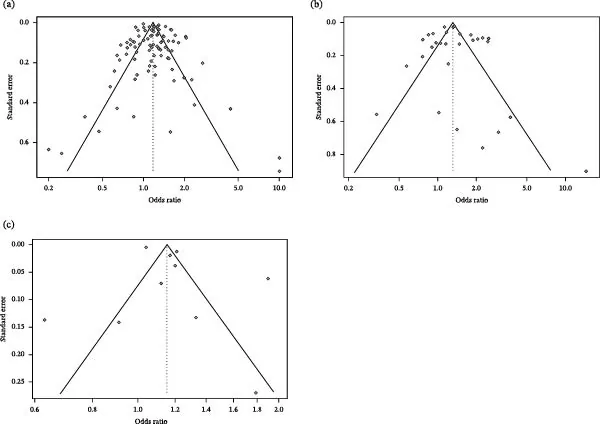
Funnel plots for publication bias in studies on nonalcoholic beverages and (a) depression, (b) anxiety, and (c) stress.

Meta‐regression analyses indicated that neither the proportion of male participants (*β* = 0.0683, *z* = 0.5596, *p* = 0.5757) nor study quality score (*β* = −0.3308, *z* = −1.9419, *p* = 0.0522) significantly influenced the effect estimates for depression (Table 2). Sensitivity analysis confirmed the robustness of these findings (OR [95% CI]: 1.18 [1.11–1.26], *p* < 0.001, *I* ^2^ = 93.7%) (Supporting Information Figure S4). Egger’s test did not suggest significant publication bias (*p* = 0.1126) (Figure 3).

**Table 2:** Meta‐regression analyses of male proportion and quality score on the association between nonalcoholic beverage consumption and risk of depression, anxiety, and stress.

Variable	Outcome	No. of studies	Coefficient	Standard error	95% lower	95% upper	*z*‐Value	*p*‐Value
Male proportion	Depression	87	0.0683	0.1220	−0.1709	0.3075	0.5596	0.5757
	Anxiety	26	0.0886	0.3955	−0.6866	0.8637	0.224	0.8227
	Stress	10	−0.3695	0.4356	−1.2232	0.4842	−0.8483	0.3963
Quality score	Depression	92	−0.3308	0.1043	−0.6647	0.0031	−1.9419	0.0522
	Anxiety	29	−1.1862	0.5885	−2.3397	−0.0327	−2.0155	**0.0439**
	Stress	11	0.3753	0.5959	−0.7926	1.5431	0.6298	0.5289

*Note:* Bold values indicate statistically significant results (*p* < 0.05).

### 3.3. Nonalcoholic Beverages Consumption and Risk of Anxiety

Meta‐analysis of 19 studies (29 reports) indicated a significant positive association between nonalcoholic beverage consumption and anxiety (OR [95% CI]: 1.31 [1.12–1.55], *p* < 0.001), with high heterogeneity (*I* ^2^ = 91.7%) (Supporting Information Figure S5). Both cross‐sectional studies (OR [95% CI]: 1.31 [1.10–1.55], *p* < 0.001) and the single cohort study (RR [95% CI]: 1.48 [1.10–1.55]) showed consistent direction of effect (Table 1).

Subgroup analyses revealed stronger associations in studies with larger sample sizes (≥5000) (OR [95% CI]: 1.61 [1.30–1.98], *p* < 0.001), those conducted in the East Asia and Pacific region (OR [95% CI]: 1.45 [1.15–1.83], *p* < 0.001), high‐income countries (OR [95% CI]: 1.28 [1.03–1.59], *p* < 0.001), pediatric‐only populations (OR [95% CI]: 1.72 [1.38–2.14], *p* < 0.001), and consumers of bubble tea and carbonated beverages (Figure 2). Statistically significant associations were observed in both males (OR [95% CI] 1.70 [1.37–2.12], *p* < 0.001) and females (OR [95% CI] 1.34 [1.01–1.78], *p* < 0.001) (Supporting Information Figure S6).

Meta‐regression identified study quality score as a significant moderator of heterogeneity (*β* = –1.1862, *z* = –2.0155, *p* = 0.0439), indicating that higher quality scores were associated with lower anxiety association estimates. The proportion of male participants was not a significant effect modifier (*β* = 0.0886, *z* = 0.2240, *p* = 0.8227; Table 2). Sensitivity analysis supported the robustness of these results (OR [95% CI]: 1.31 [1.12–1.55], *p* < 0.001, *I* ^2^ = 91.7%) (Supporting Information Figure S7). No significant publication bias was detected by Egger’s test (*p* = 0.640) (Figure 3).

### 3.4. Nonalcoholic Beverages Consumption and Risk of Stress

In 11 cross‐sectional studies, the pooled association between nonalcoholic beverage consumption and stress risk was not statistically significant (OR [95% CI]: 1.15 [0.98–1.35]), despite high heterogeneity among studies (*I* ^2^ = 96.5%) (Supporting Information Figure S8).

Subgroup analyses showed non‐significant associations in both males [OR (95% CI): 1.01 (0.84–1.20), *p* = 0.017] and females (OR [95% CI]: 1.19 [1.18–1.20], *p* = 0.220) (Supporting Information Figure S9). Significant positive associations were observed in high‐income countries (OR [95% CI]: 1.23 [1.07–1.43], *p* < 0.001), pediatric‐only populations (OR [95% CI]: 1.27 [1.04–1.54], *p* < 0.001, *I* ^2^ = 98.4%), and consumers of soft drinks (OR [95% CI]: 1.13 [1.00–1.26], *p* < 0.001). Beverage type subgroups differed significantly (*p* < 0.05) (Figure 2).

Meta‐regression showed that neither male proportion (*β* = –0.3695, *z* = –0.8483, *p* = 0.3963) nor quality score (*β* = 0.3753, *z* = 0.6298, *p* = 0.5289) significantly explained heterogeneity in stress‐related outcomes (Table 2). The corresponding bubble plots visualizing these meta‐regression relationships are presented in Supporting Information Figure S11. Sensitivity analysis excluding Sangsefidi et al. [10] substantially reduced heterogeneity and rendered the overall association statistically significant (OR [95% CI]: 1.21 [1.06,1.38], *p* = 0.037) (Supporting Information Figure S10). Egger’s test did not indicate publication bias (*p* = 0.127) (Figure 3).

## 4. Discussion

This meta‐analysis is the first to comprehensively evaluate the association between nonalcoholic beverage consumption and depression, anxiety, and stress simultaneously. Based on data from 59 original studies, 132 independent reports, and over 2 million participants, we found that nonalcoholic beverage intake was significantly positively associated with depression and anxiety. Although the overall association with stress was not statistically significant, it became significant after excluding one study that contributed substantial heterogeneity. These findings provide observational evidence for understanding how dietary factors may relate to mental health.

At the level of beverage subcategories, this study revealed distinct associations between specific types of nonalcoholic beverages and mental health outcomes. The inverse association for coffee observed in our analysis is consistent with the findings of Min et al. [6], whose dose–response analysis further indicated that moderate coffee intake was associated with the lowest levels of depression and anxiety. Conversely, sugar‐sweetened beverages, bubble tea, carbonated beverages, soft drinks, artificially sweetened beverages, and energy drinks were all positively associated with depression. This pattern of findings is consistent with several previous meta‐analyses [19, 22, 32, 33]. Specifically, two meta‐analyses have reported associations between sugar‐sweetened beverages and depression [19, 32]. One study indicated that soft drinks may elevate the risk of depression [22]. Regarding the protective effect of coffee, a meta‐analysis incorporating 11 observational studies provided robust evidence, demonstrating an inverse association between coffee intake and depression [33]. Multiple studies have shown a negative correlation between coffee intake and the risk of depression [11, 34–36]. Furthermore, the positive association between bubble tea consumption and anxiety provides support with a larger sample size for the earlier cross‐sectional findings by Wu et al. [12].

Compared to previous research, our analysis further elucidates the heterogeneity of these associations across different populations. Adolescent populations demonstrated the highest association estimates for depression, anxiety, and stress. This finding is consistent with studies conducted among South Korean adolescents [16] and may be linked to the ongoing maturation of the prefrontal cortex during adolescence, which heightens sensitivity to the neurobehavioral effects of beverage components [37]. The highest effect sizes for depression were observed in South Asia (OR = 2.15) and lower‐middle‐income countries (OR = 2.15). This aligns with the strong association (OR = 2.10) reported among Iranian adults [10, 38, 39], highlighting the complex influence of socioeconomic factors such as nutritional transition and accessibility of mental health resources [40]. The stronger association observed in male populations may be related to sex‐specific metabolic profiles or behavioral patterns [41, 42]. Data collected during the COVID‐19 period indicated a peak effect (OR = 1.61). Although the CIs were wide due to the limited number of available studies [43–45], this finding is consistent with the broader context of altered beverage consumption patterns and heightened psychosocial stress during the pandemic [46].

However, methodological variations across studies may contribute to inconsistencies in the strength of the associations. Notably, the stronger association observed for depressive symptoms compared to clinically diagnosed depression suggests that variations in depression definitions represent a significant source of heterogeneity across studies. This pattern may also indicate that nonalcoholic beverage intake has a more pronounced influence on subjective symptom experiences than that on the onset of clinically defined disorders. For anxiety, the association was stronger in East Asia and the Pacific region. This supports the findings of Cho et al. [17] regarding the association between high‐caffeine drinks and anxiety among South Korean adolescents [17], but our higher effect estimates suggest the potential influence of region‐specific factors. For stress, although the associations in gender‐specific subgroup analyses did not reach statistical significance at the individual study level, the higher effect size observed in females (female OR = 1.19; male OR = 1.01) resonates with the findings of Michels [47], which suggested greater sensitivity to diet‐related mood changes in females. Future studies with larger sample sizes are needed to explore the role of gender and underlying mechanisms [48, 49].

From a biological mechanism perspective, the inverse association for coffee may be partially explained by its complex profile of bioactive compounds, including phenolic compounds, alkaloids, and melanoidins [50]. Specifically, the effect is likely mediated by the anti‐neuroinflammatory and antioxidant properties of bioactive components such as chlorogenic acid [5, 33, 34]. In contrast, the glycemic fluctuations and insulin resistance induced by sugar‐sweetened beverage consumption may impair neuroplasticity by affecting brain‐derived neurotrophic factor (BDNF) levels and activating inflammatory pathways [51, 52]. It is noteworthy that mechanistic explanations must consider the divergence between short‐term and long‐term effects. While some studies suggest that sugar intake may produce transient mood‐improving effects, our data indicate a negative long‐term cumulative effect, consistent with the findings that long‐term cola consumption increases oxidative stress [53]. Additionally, dysfunction of the hypothalamic–pituitary–adrenal (HPA) axis is another key mechanism, whereby high‐sugar diets can disrupt normal cortisol rhythms, leading to dysregulation of the stress response system [54, 55].

The strengths of this systematic review include a comprehensive search strategy across multiple major medical databases, a rigorous dual‐independent screening and data extraction process to ensure quality, and the high statistical power afforded by the large sample size. Furthermore, the inclusion of studies from diverse global regions enhances the representativeness and generalizability of our findings across different populations. However, several limitations should be acknowledged: First, the predominance of cross‐sectional studies among the included literature limits the certainty of the causal inference. Second, residual confounding by socioeconomic status, smoking, overall diet quality, physical activity, sleep, and other lifestyle factors cannot be excluded. Third, the reliance on self‐reported beverage consumption may introduce a recall bias. Fourth, heterogeneity may have been influenced by variations in the assessment methods and categorization of beverage intake across the original studies. Additionally, despite observing risk signals for specific beverage types, the constraints of the original data prevented a more in‐depth analysis of the independent effects of specific components (e.g., specific types of sugars and additives). Future research should employ more standardized and detailed exposure assessment methods to elucidate the underlying mechanisms.

## 5. Conclusion

In conclusion, this meta‐analysis demonstrates that nonalcoholic beverage consumption is significantly associated with depression and anxiety, while the association with stress was not statistically significant in the main analysis and became significant only in the sensitivity analysis. Carbonated beverage and bubble tea consumption were significantly associated with depression and anxiety, while soft drink intake was positively associated with stress in the subgroup analysis. In contrast, coffee consumption was inversely associated with depression. These associations exhibited stronger effect sizes among pediatric, male, populations in the East Asia and Pacific region and lower‐middle‐income countries. These findings may help inform future mental health‐related dietary research and public health strategies, particularly regarding the assessment of beverage consumption patterns, while further prospective and interventional studies are needed before specific dietary recommendations can be made.

**Nomenclature**
OR: Odds ratio
RR: Risk ratio
HR: Hazard ratio
CI: Confidence interval
BDNF: Brain‐derived neurotrophic factor
HPA: Hypothalamic–pituitary–adrenal.

## Author Contributions

**Yixin Ouyang**: writing – review and editing, writing – original draft, visualization, methodology, investigation, formal analysis. **Jingyi Liang**: formal analysis. **Xijie He, Xiangrui Song, Ting Qi, Mengkun Li, Yuan Feng** **, Songyu Wu, Qianlu Ding, Zhouyang Sun, and Tingyi Jia**: writing – review and editing. **Changgui Kou and Wei Bai**: writing – review and editing, supervision, project administration, methodology, conceptualization.

## Funding

This study was supported by the 2026 "Bethune Plan" Project of the Bethune Medical Department (Grant 2026B36).

## Disclosure

All authors read and approved the final draft of the manuscript.

## Consent

The authors have nothing to report.

## Conflicts of Interest

The authors declare no conflicts of interest.

## Supporting Information

Additional supporting information can be found online in the Supporting Information section.

## Supporting information


**Supporting Information** Table S1: PRISMA 2020 checklist. Table S2: Search strategy. Table S3: Characteristics of included studies. Table S4: Quality assessment of included cross‐sectional studies. Table S5: Quality assessment of included cohort studies. Figure S1: Global distribution of odds ratios for nonalcoholic beverage consumption and mental health (depression, anxiety, or stress). Figure S2: Forest plot of the association between nonalcoholic beverage consumption and depression risk. Figure S3: Forest plot of gender‐specific associations of nonalcoholic beverages with depression. Figure S4: Sensitivity analysis of the association between nonalcoholic beverage consumption and depression risk. Figure S5: Forest plot of the association between nonalcoholic beverage consumption and anxiety risk. Figure S6: Forest plot of gender‐specific associations of nonalcoholic beverages with anxiety. Figure S7: Sensitivity analysis of the association between nonalcoholic beverage consumption and anxiety risk. Figure S8: Forest plot of the association between nonalcoholic beverage consumption and stress risk. Figure S9: Forest plot of gender‐specific associations of nonalcoholic beverages with stress. Figure S10: Sensitivity analysis of the association between nonalcoholic beverage consumption and stress risk. Figure S11: Meta‐regression plots of male proportion and quality score on the association between nonalcoholic beverages and depression, anxiety, and stress.

## Data Availability

Research data are not shared.
