# Supplementary material for: Nonalcoholic Beverage Consumption and the Risk of Depression, Anxiety, and Stress: A Systematic Review and Meta‐Analysis
Source: Depress Anxiety. 2026 Aug 31;2026:7404084. doi: 10.1155/da/7404084 (PMC13527640; doi:10.1155/da/7404084)
Supplement: Supplementary file 1 [file DA-2026-7404084-s001.docx]

**Non-alcoholic Beverage Consumption and the Risk of Depression, Anxiety and Stress: A Systematic Review and Meta-analysis**

Supplementary Data:

1. Table S1. PRISMA 2020 checklist
2. Table S2. Search strategy
3. Table S3. Characteristics of included studies.
4. Table S4. Quality assessment of included cross-sectional studies
5. Table S5. Quality assessment of included cohort studies
6. Fig. S1. Global distribution of odds ratios for non-alcoholic beverage consumption and mental health (depression, anxiety, or stress)
7. Fig. S2. Forest plot of the association between non-alcoholic beverage consumption and depression risk
8. Fig. S3. Forest plot of gender-specific associations of non-alcoholic beverages with depression
9. Fig. S4. Sensitivity analysis of the association between non-alcoholic beverage consumption and depression risk
10. Fig. S5. Forest plot of the association between non-alcoholic beverage consumption and anxiety risk
11. Fig. S6. Forest plot of gender-specific associations of non-alcoholic beverages with anxiety
12. Fig. S7. Sensitivity analysis of the association between non-alcoholic beverage consumption and anxiety risk
13. Fig. S8. Forest plot of the association between non-alcoholic beverage consumption and stress risk
14. Fig. S9. Forest plot of gender-specific associations of non-alcoholic beverages with stress
15. Fig. S10. Sensitivity analysis of the association between non-alcoholic beverage consumption and stress risk
16. Fig. S11. Meta-regression plots of male proportion and quality score on the association between non-alcoholic beverages and depression, anxiety, stress
17. Included references of meta-analysis

Table S1. PRISMA 2020 checklist

| **Section and Topic** | **Item #** | **Checklist item** | **Location where item is reported** |
| --- | --- | --- | --- |
| **TITLE** | | |  |
| Title | 1 | Identify the report as a systematic review. | Page1 |
| **ABSTRACT** | | |  |
| Abstract | 2 | See the PRISMA 2020 for Abstracts checklist. | Page2-3 |
| **INTRODUCTION** | | |  |
| Rationale | 3 | Describe the rationale for the review in the context of existing knowledge. | Page4-5 |
| Objectives | 4 | Provide an explicit statement of the objective(s) or question(s) the review addresses. | Page4-5 |
| **METHODS** | | |  |
| Eligibility criteria | 5 | Specify the inclusion and exclusion criteria for the review and how studies were grouped for the syntheses. | Page5-6 |
| Information sources | 6 | Specify all databases, registers, websites, organisations, reference lists and other sources searched or consulted to identify studies. Specify the date when each source was last searched or consulted. | Page5-6 |
| Search strategy | 7 | Present the full search strategies for all databases, registers and websites, including any filters and limits used. | Page5-6& Table S2 |
| Selection process | 8 | Specify the methods used to decide whether a study met the inclusion criteria of the review, including how many reviewers screened each record and each report retrieved, whether they worked independently, and if applicable, details of automation tools used in the process. | Page5-6 |
| Data collection process | 9 | Specify the methods used to collect data from reports, including how many reviewers collected data from each report, whether they worked independently, any processes for obtaining or confirming data from study investigators, and if applicable, details of automation tools used in the process. | Page6-7 |
| Data items | 10a | List and define all outcomes for which data were sought. Specify whether all results that were compatible with each outcome domain in each study were sought (e.g. for all measures, time points, analyses), and if not, the methods used to decide which results to collect. | Page6-7 |
|  | 10b | List and define all other variables for which data were sought (e.g. participant and intervention characteristics, funding sources). Describe any assumptions made about any missing or unclear information. | Page6 |
| Study risk of bias assessment | 11 | Specify the methods used to assess risk of bias in the included studies, including details of the tool(s) used, how many reviewers assessed each study and whether they worked independently, and if applicable, details of automation tools used in the process. | Page7-8 |
| Effect measures | 12 | Specify for each outcome the effect measure(s) (e.g. risk ratio, mean difference) used in the synthesis or presentation of results. | Page7 |
| Synthesis methods | 13a | Describe the processes used to decide which studies were eligible for each synthesis (e.g. tabulating the study intervention characteristics and comparing against the planned groups for each synthesis (item #5)). | Page7-8 |
|  | 13b | Describe any methods required to prepare the data for presentation or synthesis, such as handling of missing summary statistics, or data conversions. | Page7-8 |
|  | 13c | Describe any methods used to tabulate or visually display results of individual studies and syntheses. | Page7-8 |
|  | 13d | Describe any methods used to synthesize results and provide a rationale for the choice(s). If meta-analysis was performed, describe the model(s), method(s) to identify the presence and extent of statistical heterogeneity, and software package(s) used. | Page8 |
|  | 13e | Describe any methods used to explore possible causes of heterogeneity among study results (e.g. subgroup analysis, meta-regression). | Page8 |
|  | 13f | Describe any sensitivity analyses conducted to assess robustness of the synthesized results. | Page8 |
| Reporting bias assessment | 14 | Describe any methods used to assess risk of bias due to missing results in a synthesis (arising from reporting biases). | Page8 |
| Certainty assessment | 15 | Describe any methods used to assess certainty (or confidence) in the body of evidence for an outcome. | Page8 |
| **RESULTS** | | |  |
| Study selection | 16a | Describe the results of the search and selection process, from the number of records identified in the search to the number of studies included in the review, ideally using a flow diagram. | Page9 & Fig.1 |
|  | 16b | Cite studies that might appear to meet the inclusion criteria, but which were excluded, and explain why they were excluded. | Page9 & Fig.1 |
| Study characteristics | 17 | Cite each included study and present its characteristics. | Page9 & Table 1 |
| Risk of bias in studies | 18 | Present assessments of risk of bias for each included study. | Table S4 & Table S5 |
| Results of individual studies | 19 | For all outcomes, present, for each study: (a) summary statistics for each group (where appropriate) and (b) an effect estimate and its precision (e.g. confidence/credible interval), ideally using structured tables or plots. | Table 1 & Fig.S1-10 |
| Results of syntheses | 20a | For each synthesis, briefly summarise the characteristics and risk of bias among contributing studies. | Page9-11 & Table 1 |
|  | 20b | Present results of all statistical syntheses conducted. If meta-analysis was done, present for each the summary estimate and its precision (e.g. confidence/credible interval) and measures of statistical heterogeneity. If comparing groups, describe the direction of the effect. | Page9-11 |
|  | 20c | Present results of all investigations of possible causes of heterogeneity among study results. | Page9-11 & Fig.2 |
|  | 20d | Present results of all sensitivity analyses conducted to assess the robustness of the synthesized results. | Page9-11 & Fig.S3, S6, S9 |
| Reporting biases | 21 | Present assessments of risk of bias due to missing results (arising from reporting biases) for each synthesis assessed. | Page9-11 & Fig.3 |
| Certainty of evidence | 22 | Present assessments of certainty (or confidence) in the body of evidence for each outcome assessed. | Page9-11 |
| **DISCUSSION** | | |  |
| Discussion | 23a | Provide a general interpretation of the results in the context of other evidence. | Page11-14 |
|  | 23b | Discuss any limitations of the evidence included in the review. | Page11-14 |
|  | 23c | Discuss any limitations of the review processes used. | Page11-14 |
|  | 23d | Discuss implications of the results for practice, policy, and future research. | Page11-14 |
| **OTHER INFORMATION** | | |  |
| Registration and protocol | 24a | Provide registration information for the review, including register name and registration number, or state that the review was not registered. | Page5 |
|  | 24b | Indicate where the review protocol can be accessed, or state that a protocol was not prepared. | Page5-6 |
|  | 24c | Describe and explain any amendments to information provided at registration or in the protocol. | Page5-6 |
| Support | 25 | Describe sources of financial or non-financial support for the review, and the role of the funders or sponsors in the review. | Page16 |
| Competing interests | 26 | Declare any competing interests of review authors. | Page16 |
| Availability of data, code and other materials | 27 | Report which of the following are publicly available and where they can be found: template data collection forms; data extracted from included studies; data used for all analyses; analytic code; any other materials used in the review. | TableS3 |

*From:* Page MJ, McKenzie JE, Bossuyt PM, Boutron I, Hoffmann TC, Mulrow CD, et al. The PRISMA 2020 statement: an updated guideline for reporting systematic reviews. BMJ 2021;372:n71. doi: 10.1136/bmj.n71

For more information, visit: <http://www.prisma-statement.org/>

Table S2. Search strategy

| PubMed  (Title/Abstract) | ("Coffee"[Title/Abstract] OR "Tea"[Title/Abstract] OR "green tea"[Title/Abstract] OR "black tea"[Title/Abstract] OR "Beverages"[Title/Abstract] OR "beverage"[Title/Abstract] OR "soft drink"[Title/Abstract] OR "carbonated beverages"[Title/Abstract]) AND ("depress*"[Title/Abstract] OR "Depression"[Title/Abstract] OR "Depressive"[Title/Abstract] OR "Depressed"[Title/Abstract] OR "depressive symptom"[Title/Abstract] OR "emotional depression"[Title/Abstract] OR "depression disorder"[Title/Abstract] OR "depression risk"[Title/Abstract] OR "anxiety"[MeSH Terms] OR "anxiety*"[Title/Abstract] OR "Anxieties"[Title/Abstract] OR "stress*"[Title/Abstract]) AND ("observational study"[Publication Type] OR "observational studies as topic"[MeSH Major Topic] OR "longitudinal studies"[MeSH Major Topic] OR "prospective studies"[MeSH Major Topic] OR "cohort studies"[MeSH Major Topic] OR "cohort*"[Title/Abstract] OR "prospective*"[Title/Abstract] OR "retrospective*"[Title/Abstract] OR "longitudinal"[Title/Abstract] OR "Observational"[Title/Abstract] OR "follow-up"[Title/Abstract] OR "nested case control"[Title/Abstract] OR "case-cohort"[Title/Abstract] OR "case-control"[Title/Abstract] OR "relative risk"[Title/Abstract] OR "hazard ratio"[Title/Abstract] OR "odds ratio"[Title/Abstract] OR "risk"[Title/Abstract]) |
| --- | --- |
| Web of Science  (Topic) | ("Coffee" OR "Tea" OR "Green tea" OR "Black tea" OR "beverages" OR "beverage" OR "soft drink" OR "Carbonated Beverages") AND ("Depress* " OR "Depression" OR "Depressive" OR "Depressed" OR "Depressive symptom" OR "Emotional depression" OR "Depression disorder" OR "Depression risk" OR "Anxiety" OR "Anxiety*" OR "Anxieties" OR "Stress*") AND ("Observational Study" OR "Observational Studies as topic" OR "Longitudinal Studies" OR "Prospective Studies" OR "Cohort Studies" OR "cohort*" OR "prospective*" OR "retrospective*" OR "longitudinal" OR "observational" OR "follow-up" OR "nested case-control" OR "case-cohort" OR "case control" OR "relative risk" OR "hazard ratio" OR " odds ratio" OR " risk" ) |
| APA PsycInfo (Abstract) | AB ("Coffee" OR "Tea" OR "green tea" OR "black tea" OR "Beverages" OR "beverage" OR "soft drink" OR "carbonated beverages") AND ( " depress*" OR "Depression" OR "Depressive" OR "Depressed" OR "depressive symptom" OR "emotional depression" OR "depression disorder" OR "depression risk" OR "anxiety" OR "anxiety*" OR "Anxieties" OR "stress*") AND ("observational study" OR "observational studies as topic" OR "longitudinal studies" OR "prospective studies" OR "cohort studies" OR "cohort*" OR "prospective*" OR "retrospective*" OR "longitudinal" OR "Observational" OR "follow-up" OR "nested case control" OR "case-cohort" OR "case-control" OR "relative risk" OR "hazard ratio" OR "odds ratio" OR "risk" ) |
| Embase (Article  title, Abstract) | ('coffee':ab,ti OR 'tea':ab,ti OR 'green tea':ab,ti OR 'black tea':ab,ti OR 'beverages':ab,ti OR 'beverage':ab,ti OR 'soft drink':ab,ti OR 'carbonated beverages':ab,ti) AND ('depress*':ab,ti OR 'depression':ab,ti OR 'depressive':ab,ti OR 'depressed':ab,ti OR 'depressive symptom':ab,ti OR 'emotional depression':ab,ti OR 'depression disorder':ab,ti OR 'depression risk':ab,ti OR 'anxiety':ab,ti OR 'anxiety*':ab,ti OR 'anxieties':ab,ti OR 'stress*':ab,ti) AND ('observational study':ab,ti OR 'observational studies as topic':ab,ti OR 'longitudinal studies':ab,ti OR 'prospective studies':ab,ti OR 'cohort studies':ab,ti OR 'cohort*':ab,ti OR 'prospective*':ab,ti OR 'retrospective*':ab,ti OR 'longitudinal':ab,ti OR 'observational':ab,ti OR 'follow up':ab,ti OR 'nested case-control':ab,ti OR 'case cohort':ab,ti OR 'case control':ab,ti OR 'relative risk':ab,ti OR 'hazard ratio':ab,ti OR 'odds ratio':ab,ti OR 'risk':ab,ti) |

| Table S3. Characteristics of included studies. | | | | | | | | | | | | | | | | | | | | | | | | |
| --- | --- | --- | --- | --- | --- | --- | --- | --- | --- | --- | --- | --- | --- | --- | --- | --- | --- | --- | --- | --- | --- | --- | --- | --- |
| Article | X | Y | Study name | Study site | Survey time | Study design | Sampling method | Sample size | Depression | | Anxiety | | Stress | | General population | | | Cases | | | | | Quality score | Quality grade |
|  |  |  |  |  |  |  |  |  | Scale | Cut-off | Scale | Cut-off | Scale | Cut-off | Inclusion criteria for age | Mean age | Male proportion | Number | Mean score | Score SD | Mean age | Male proportion |  |  |
| Yang et al.-a (2024)(1) | sugar-sweetened beverages | depressive symptoms | NR | China | NR | cross-sectional study | stratified random whole cluster sampling | 6,259 | the Self-rating Depression Scale (SDS) | ≥53 | NR | NR | NR | NR | 19-22 | 20.18 (1.03) | 43.86% | 2,723 | NR | NR | NR | NR | 6 | moderate |
| Yang et al.-b (2024)(2) | bubble tea | depressive symptoms, anxiety symptoms | baseline data from the Nurses' Mental Health Study (NMHS) | China | NR | cross-sectional study | NR | 132,910 | PHQ-9 | ≥5 | GAD-7 | ≥5 | NR | NR | NR | Median: 34 | 6.52% | NR | NR | NR | NR | NR | 6 | moderate |
| Tang et al. (2024)(3) | carbonated beverages, coffee, tea | depressive symptoms, anxiety symptoms | NR | China | between February and April 2024 | cross-sectional study | convenience sampling method | 142 | PHQ-9 | ≥10 | GAD-7 | ≥ 10 | NR | NR | ≥18 | 20 (1.7) | 37.30% | NR | NR | NR | NR | NR | 5 | moderate |
| Qiu et al. (2024)(4) | sugar-sweetened beverages | anxiety symptoms | The Japan Public Health Center-based Prospective Study for the Next Generation (JPHC-NEXT) | China | NR | cross-sectional study | random coded sampling | 3,026 | NR | NR | SAS | ≥50 | NR | NR | 19-22 | NR | 49.27% | 813 | NR | NR | NR | 47.60% | 6 | moderate |
| Liu et al. (2024)(5) | coffee | depressive symptoms | NR | China | between June and August 2023 | longitudinal study | multi-stage, stratified cluster random sampling | 10,770 | PHQ-9 | ≥10 | NR | NR | NR | NR | 14-18 | 15.89 (0.60) | 51.60% | 1,460 | NR | NR | 15.94 (0.63) | 44.25% | 5 | moderate |
| Li et al. (2024)(6) | sugary drinks | depressive symptoms | NR | China | between January 2018 and December 2020 | cross-sectional study | convenience sampling method | 118,723 | PHQ-9 | ≥10 | NR | NR | NR | NR | NR | NR | 65.60% | 37,411 | NR | NR | NR | 62.25% | 6 | moderate |
| Ikram et al. (2024)(7) | carbonated soft drink | depressive symptoms, anxiety symptoms | NR | Pakistan | NR | cross-sectional study | nonprobability sampling technique | 500 | PHQ-9 | ≥5 | GAD-7 | ≥5 | NR | NR | 18-30 | NR | 41.60% | depression: 407; anxiety:388 | NR | NR | NR | NR | 4 | moderate |
| Cho et al. (2024)(8) | high-caffeine drink; sweet drink | anxiety symptoms | the Korea Youth Risk Behavior Web-based Survey (KYRBS) 2022 | Korea | 2022 | cross-sectional study | stratified sampling | 46,873 | NR | NR | GAD-7 | ≥5 | NR | NR | 12-18 | NR | 50.51% | 16,560 | NR | NR | NR | 41.79% | 4 | moderate |
| Chen et al. (2024)(9) | sugary beverages; artificially sweetened beverages; natural juices | depression disorder | UK Biobank | United Kingdom | 2006-2010baseline data-2021.9.30 | prospective cohort study | NR | 180,599 | NR | hospital primary/ secondary diagnosis or death primary and contributory +ICD-10 | NR | NR | NR | NR | 37-73 | Median: 58 (50-63) | 45.80% | 4,915 | NR | NR | Median: 58 (50-63) | 36.80% | 8 | high |
| Bahrami et al. (2024)(10) | soft drinks; artificial juice; sugar-sweetened beverages | depressive symptoms, anxiety symptoms | The LIPOKAP study | Islamic Republic of Iran | between February 2018 and July 2019 | cross-sectional study | stratified multistage random cluster sampling | 1,970 | Hospital Anxiety and Depression Scale (HADS) | ≥8 | Hospital Anxiety and Depression Scale (HADS) | ≥8 | NR | NR | ≥18 | 39.8 (13.9) | NR | NR | NR | NR | NR | NR | 6 | moderate |
| Zhang et al. (2023)(11) | sugar-sweetened beverages | depressive symptoms, anxiety symptoms | NR | China | February 2020 and May 2020 | cross-sectional study | cluster sampling | 15,936 | PHQ-9 | ≥5 | GAD-7 | ≥5 | NR | NR | College Students | 20.58 (1.81) | 42.00% | 3,804 | NR | NR | NR | 42.82% | 4 | moderate |
| Yin et al. (2023)(12) | caffeine intake | depressive symptoms | National Health and Nutrition Examination Survey (NHANES) | United States | between 2005 and 2018 | cross-sectional study | stratified, multistage probability cluster sampling | 34,697 | PHQ-9 | ≥10 | NR | NR | NR | NR | ≥18 | NR | 49.25% | 2,988 | 14.06 | 0.16 | NR | 37.35% | 8 | high |
| Sahril et al. (2023)(13) | carbonated soft drink | depressive symptoms | National School-Based Health Survey 2022 | Malaysia | June to July 2022 | cross-sectional study | multistage stratified cluster sampling | 33,523 | PHQ-9 | ≥10 | NR | NR | NR | NR | 13-17 | NR | 46.20% | 9,103 | NR | NR | NR | 29.46% | 6 | moderate |
| Min et al. (2023)(14) | coffee | depressive symptoms, anxiety symptoms | UK Biobank | United Kingdom | Between April 2006 and December 2010 | prospective cohort study | NR | 146,566 | PHQ-9 | ≥10 | GAD-7 | ≥10 | NR | NR | 37-73 | 55.9 (7.7) | 43.54% | depression:15,759; anxiety:6,582 | NR | NR | NR | NR | 7 | high |
| Luo et al. (2023)(15) | coffee, sugar-sweetened beverages | depressive symptoms | health management center of the third xiangya hospital, Central South University | China | between January 1, 2015, and December 31, 2020 | cross-sectional study | convenience sampling | 48,637 | PHQ-9 | ≥10 | NR | NR | NR | NR | 42-52 | NR | 0 | 11,413 | NR | NR | NR | 0 | 4 | moderate |
| Wu et al. (2022)(16) | bubble tea | depressive symptoms, anxiety symptoms | NR | China | NR | cross-sectional study | NR | 735 | PHQ-2 | ≥3 | GAD-2 | ≥3 | NR | NR | 18-<30 | 24 | 64% | depression:123; anxiety:80 | NR | NR | NR | depression:63.41%; anxiety:71.25% | 2 | low |
| Ra (2022)(17) | sugar-sweetened beverages | depressive symptoms, stress | the Korea Youth Risk Behavior Web-based Survey (KYRBS)2021 | Korea | 2021 | cross-sectional study | stratified sampling | 24,004 | NR | Single-item measure | NR | NR | NR | Single-item measure | 12-18 | NR | NR | depressive symptoms: 6,579; stress:19,806 | NR | NR | NR | NR | 7 | moderate |
| Nouri-Majd et al. (2022)(18) | coffee and caffeine | depressive symptoms, anxiety symptoms | Study on the Epidemiology of Psychological, Alimentary Health and Nutrition (SEPAHAN) | Islamic Republic of Iran | between April 2010 and May 2010 | cross-sectional study | multistage stratified sampling | 3,362 | HADS | ≥8 | HADS | ≥8 | NR | NR | NR | 36.2 (7.8) | 40.87% | depression:943; anxiety:448 | NR | NR | NR | NR | 7 | moderate |
| Long et al. (2022)(19) | soft/sugared drinks | stress | China Health and Nutrition Surveys (CHNS) | China | 2015 | cross-sectional study | multistage, random cluster sampling | 8,910 | NR | NR | NR | NR | Perceived Stress Scale-14 | ≥27 | 18-65 | NR | 46.70% | high:3,018 | NR | NR | NR | 45.99% | 6 | moderate |
| Liu et al.-a (2022)(20) | sugar-sweetened beverages | depressive symptoms | NR | China | 2020 | cross-sectional study | stratified cluster random sampling | 1,311 | The Children's Depression Inventory (CDI) | >19 | NR | NR | NR | NR | 7-17 | 12.07 (3.09) | 50.19% | 183 | NR | NR | NR | 11.55% | 6 | moderate |
| Liu et al.-b (2022)(21) | soft drinks | depressive symptoms | Youth Risk Behavior Survey (YRBS) | United States | 2011-2019 | cross-sectional study | multistage, cluster sampling | 73,074 | NR | Single-item measure | NR | NR | NR | NR | 13-18 | NR | 49.40% | 22,629 | NR | NR | NR | NR | 3 | low |
| Kim et al. (2022)(22) | sugar-sweetened beverages | depressive symptoms | Korean National Health and Nutrition Examination Survey (KNHANES) | Korea | 2014 and 2016 | cross-sectional study | stratified, multistage probability-sampling | 5,465 | PHQ-9 | ≥5 | NR | NR | NR | NR | ≥20 | NR | 39.71% | 1,006 | NR | NR | NR | NR | 5 | moderate |
| Fond et al. (2022)(23) | coffee | depressive symptoms, anxiety symptoms | NR | France | between April 1st 2019 and June 31st 2019. | cross-sectional study | NR | 2,003 | HAD Hamilton Anxiety Depression rating scale | ≥11 | HAD Hamilton Anxiety Depression rating scale | ≥11 | NR | NR | NR | 26 | 27.96% | depression:174; anxiety:647 | NR | NR | NR | depression:48.72%; anxiety:78.83% | 6 | moderate |
| Canas-Simião et al. (2022)(24) | soft drinks | depression disorder | NR | Portugal | from April to August 2020 (during lockdown) | cross-sectional study | nonprobabilistic snowball method | 968 | NR | reported a diagnosis of depression | NR | NR | NR | NR | >18 | NR | 21.49% | 484 | NR | NR | 50.88 (13.1) | 21.49% | 4 | moderate |
| Bi et al. (2022)(25) | sugar-sweetened beverages | depressive symptoms | NR | China | from April to May 2022 | cross-sectional study | stratified cluster sampling | 6,695 | the Self-rating Depression Scale (SDS) | ≥50 | NR | NR | NR | NR | 18-24 | NR | 42.75% | 3,080 | NR | NR | NR | 40.52% | 8 | high |
| Bharati et al. (2022)(26) | soft drinks | depressive symptoms | NR | India | from December 20, 2019 to March 21, 2020. | cross-sectional study | systematic random sampling | 838 | PHQ-9 | ≥5 | NR | NR | NR | NR | 11-19 | NR | 28.64% | 429 | NR | NR | NR | 79.25% | 8 | high |
| Bakhriansyah et al. (2022)(27) | tea | anxiety symptoms | NR | Indonesia | in December 2021 | cross-sectional study | simple random sampling | 332 | NR | NR | the Zung Self-Rating Anxiety Scale (ZSAS) | ≥45 | NR | NR | NR | 19.58 (1.43) | 29.22% | 81 | NR | NR | NR | 19.75% | 6 | moderate |
| Yim et al. (2021)(28) | carbonated beverage, caffeinated beverage | depressive symptoms; stress | the Korean Youth Risk Behavior Survey (KYRBWS) | Korea | in 2015, 2017, and 2019 | cross-sectional study | stratified, clustered, multistage probability random sampling | 187,622 | NR | Single-item measure | NR | NR | A five-point Likert scale | Single-item measure | NR | NR | 51.43% | depression:47,534; stress: NR | NR | NR | NR | NR | 5 | moderate |
| Michels et al. (2021)(29) | soft drinks | stress | The Belgian Health Interview Survey | Belgium | 2013 | cross-sectional study | multistage sampling | 4,687 | NR | NR | NR | NR | the General Health Questionnaire (GHQ12) | ≥3 | ≥15 | NR | 48.05% | 1,483 | NR | NR | NR | NR | 7 | moderate |
| Kleppang et al. (2021)(30) | sugar-sweetened beverages; artificially sweetened beverages; energy drinks | depressive symptoms | the Ungdata Survey | Norway | from 2017 to 2019 | cross-sectional study | NR | 241,730 | scale based on Hopkins Symptom Checklist 90 (SCL-90) | NR | NR | NR | NR | NR | 13-19 | NR | 48.31% | 53,873 | NR | NR | NR | 26.42% | 5 | moderate |
| Kim et al. (2021)(31) | soft drinks | depressive symptoms | Korean National Health and Nutrition Examination Survey (KNHANES) | Korea | from January to December 2016 | cross-sectional study | stratified multistage probability sampling | 3,086 | PHQ-9 | ≥10 | NR | NR | NR | NR | 18-65 | NR | 38.43% | 153 | NR | NR | NR | 28.10% | 6 | moderate |
| Kashino et al. (2021)(32) | soft drinks | depressive symptoms | The Furukawa Nutrition and Health Study | Japan | April 2012 and 2015 May 2013 and 2016 | cohort study | NR | 935 | Center for Epidemiological Studies Depression Scale [CES-D] | ≥16 | NR | NR | NR | NR | 21-71 | NR | 89.20% | 158 | NR | NR | NR | NR | 6 | moderate |
| Collins et al. (2021)(33) | sugar-sweetened beverages | depression disorder or depressive symptoms, anxiety disorder or anxiety symptoms | the Strengthening the Reporting of Observational Studies in Epidemiology (STROBE) | Australia | from July 2017 to June 2018 | cross-sectional study | stratified multistage sampling | 1,340 | NR | self-reported | NR | NR | NR | NR | 18-29 | NR | 47.39% | depression:219; anxiety:308 | NR | NR | NR | depression:35.62%; anxiety:32.14% | 7 | moderate |
| Sangsefidi et al. (2020)(34) | sweetened drinks | depressive symptoms, anxiety symptoms, stress | Yazd Health Study (YaHS) | Islamic Republic of Iran | during September 2014–March 2016 | cross-sectional study | cluster sampling; two-level clustered random sampling | 9,965 | DASS 21 | ≥10 | DASS 21 | ≥8 | DASS 21 | ≥15 | 20-70 | NR | 49.38% | depression:827; anxiety:1,279; stress:1,279 | NR | NR | NR | NR | 4 | moderate |
| Miller et al. (2020)(35) | sugar-sweetened beverages, artificially-sweetened soda, energy, sports, fruit juices | depression disorder | NR | Australia | between February and April 2017 | cross-sectional study | NR | 3,430 | NR | had ever been told by a doctor or health professional | NR | NR | NR | NR | ≥18 | 46.9 (18.3) | 51% | NR | NR | NR | NR | NR | 7 | moderate |
| Kimura et al. (2020)(36) | coffee and caffeine | depressive symptoms | NR | Japan | from April to May in 2011 and 2012 | cross-sectional study | NR | 1,992 | Center for Epidemiologic Studies Depression Scale (CES-D) | ≥16 | NR | NR | NR | NR | 65-94 | NR | 0 | 438 | NR | NR | NR | 0 | 6 | moderate |
| Khan et al. (2020)(37) | sugary drinks | depressive symptoms | NR | Bangladesh | during November 2012 to January 2013 | cross-sectional study | NR | 755 | 10-item of the Center for Epidemiologic Studies Depression Scale (CES-D-10) | ≥10 | NR | NR | NR | NR | 13-16 | 14.26 (1.15) | 49.27% | 189 | NR | NR | NR | NR | 6 | moderate |
| Sousa et al. (2019)(38) | natural juice; sugar sweetened beverages | depressive symptoms | the Brazilian National Health Survey (PNS) | Brazil | 2013 | cross-sectional study | Multistage sampling | 46,785 | PHQ-9 | ≥10 | NR | NR | NR | NR | 20-59 | NR | 47.90% | 3,556 | NR | NR | NR | NR | 4 | moderate |
| Pengpid et al.-a (2019)(39) | carbonated soft drink | anxiety symptoms | Global School-Based Student Health Survey (GSHS) | Bangladesh, Indonesia, Laos, Philippines, Thailand, and Timor-Leste | 2014-2015 | cross-sectional study | multistage, cluster sampling | 36,173 | NR | NR | NR | Single-item measure | NR | NR | 13-17 | Median: 14 (3) | 44.49% | 2,767 | NR | NR | NR | NR | 3 | low |
| Pengpid et al.-b (2019)(40) | carbonated soft drink | depressive symptoms | NR | Indonesia， Malaysia，Myanmar，Thailand， Vietnam | NR | cross-sectional study | stratified random sampling | 3,353 | 10-item of the Center for Epidemiologic Studies Depression Scale (CES-D-10) | ≥15 | NR | NR | NR | NR | 18-30 | Median: 20 (3) | 37.13% | 354 | NR | NR | NR | NR | 2 | low |
| Elstgeest et al. (2019)(41) | sugar-sweetened beverages, fruit juices | depressive symptoms | Invecchiare in Chianti (The InCHIANTI study) | Italy | 1998-2000; 2001–2003, 2004–2006 2007–2009 | prospective cohort study | multistage, stratified sampling | 1,058 | Center for Epidemiologic Studies Depression Scale (CES-D) | ≥20 | NR | NR | NR | NR | 20-102 | 65.8 (15.2) | 45.27% | 187 | NR | NR | NR | NR | 7 | high |
| Sanchez-Villegas et al. (2018)(42) | added sugars and sugar-sweetened beverages | depression disorder | the Seguimiento Universidad de Navarra (SUN) | Spain | December 1999, biennially, up to December 2016 | prospective cohort study | NR | 15,546 | NR | Clinical diagnoses | NR | NR | NR | NR | NR | 37.2 | NR | 769 | NR | NR | NR | NR | 7 | high |
| Park et al. (2018)(43) | soft drinks | depressive symptoms, stress | the 2016 Korea Youth Risk Behaviour Web-based Survey (KYRBS) | Korea | 2016 | cross-sectional study | stratified cluster sampling | 65528 | NR | NR | NR | NR | NR | the question ‘To what degree are you usually stressed?’ Participants chose from response options of (i) very much, (ii) somewhat, (iii) average, (iv) not so much, or (v) not at all. selected options 1 or 2 were classified into an ‘above average group’ and those who selected options 3–5 were classified as the ‘average or below average’ group | NR | 14.99 | 51.59% | NR | NR | NR | NR | NR | 3 | low |
| Navarro et al. (2018)(44) | coffee | depression disorder | The “Seguimiento Universidad de Navarra” (SUN) project | Spain | started in December 1999, every other year to participants to update information, by 2017 | prospective cohort study | NR | 14,413 | NR | met 2 criteria simultaneously: (a) a validated self-reported new physician-made diagnosis of depression together with (b) new-onset habitual use of antidepressants (in the previous 2 years) | NR | NR | NR | NR | NR | 36.4 (11.5) | 40.00% | 199 | NR | NR | NR | NR | 7 | high |
| Masana et al. (2018)(45) | coffee | depressive symptoms | the MEditerranean Islands Study (MEDIS study) | multi-national | 2005-2011 | cross-sectional study | convenience sampling | 2,718 | the Geriatric Depression Scale (GDS) | ≥6 | NR | NR | NR | NR | ≥65 | 74.2 (7.3) | 50% | 496 | NR | NR | NR | 43% | 4 | moderate |
| Kim et al. (2018)(46) | coffee and caffeine | depression disorder or depressive symptoms | Korean National Health and Nutrition Examination Survey (KNHANES) | Korea | in 2010, 2011, and 2012 | cross-sectional study | stratified, multistage, probability-sampling | 9,576 | NR | “Have you ever been diagnosed with depression by a physician?” and “Do you have depression at present?” (n = 171) and “Are you getting treatment for depression at present?” (n = 108) and “In the past year, have you felt sadness or despair continuously for two or more weeks that was severe enough to interfere with daily life?” (n = 1221). Participants who reported at least one “yes” were regarded as having depression. | NR | NR | NR | NR | ≥19 | NR | 40.23% | 1,443 | NR | NR | 46.2 (0.5) | 33.60% | 6 | moderate |
| Chan et al. (2018)(47) | tea | depressive symptoms, anxiety symptoms | The Diet and Healthy Aging Study (DaHA) | Singapore | in July 2011 | cross-sectional study | NR | 614 | the 15-item Geriatric Depression Scale (GDS-15) | NR | the 20-item Geriatric Anxiety Inventory (GAI) | NR | NR | NR | 60-93 | Median: 67 | 29.80% | depression:366; anxiety:181 | GDS: Median: 1; GAI: Median: 0 | NR | NR | NR | 5 | moderate |
| Yu et al. (2017)(48) | coffee, artificially sweetened beverages | major depression disorder | the Atlantic Partnership for Tomorrow’s Health cohort study (Atlantic PATH) | Canada | 2009-2013 | cross-sectional study | NR | 18,838 | NR | NR | NR | NR | NR | NR | 35-69 | NR | 31.08% | 3,217 | NR | NR | NR | 20.39% | 6 | moderate |
| Kim et al. (2017)(49) | energy drink | stress | the Korea Youth Risk Behavior Web-based Survey (KYRBS) | Korea | 2014-2015 | cross-sectional study | stratified, two-stage clustered sampling | 121,106 | NR | NR | NR | NR | NR | severe, moderate, mild, a little, and no stress | 13-18 | NR | 50.12% | NR | NR | NR | NR | NR | 6 | moderate |
| Yu et al. (2015)(50) | soft drinks | depressive symptoms | The Tianjin Chronic Low-grade Systemic Inflammation and Health (TCLSIH or TCLSIHealth) Cohort Study | China | 2007 | cross-sectional study | NR | 3,667 | the Zung Self-Rating Depression Scale (SDS) | ≥50 | NR | NR | NR | NR | NR | 42.5 (12.1) | 60.20% | 280 | NR | NR | NR | NR | 5 | moderate |
| Zahedi et al. (2014)(51) | sweetened beverages (soda, soft drinks) | depressive symptoms, anxiety symptoms | CASPIAN-IV (Childhood and Adolescence Surveillance and Prevention of Adult Non communicable Disease) study | Islamic Republic of Iran | 2011-2012 | cross-sectional study | stratified cluster sampling | 13,486 | NR | Single-item measure | NR | Single-item measure | NR | NR | 6-18 | 12.47 (3.36) | 50.76% | 2,794 | NR | NR | NR | 46.39% | 5 | moderate |
| Trapp et al. (2014)(52) | energy drink | depressive symptoms, anxiety symptoms, stress | the Western Australian Pregnancy Cohort (Raine) Study | Australia | between 1989 and 1991，20-year cohort follow-up | cross-sectional study | NR | 1,062 | DASS 21 | ≥10 | DASS 21 | ≥8 | DASS 21 | ≥15 | NR | 19.98 (3.29) | 47.27% | NR | NR | NR | NR | NR | 9 | high |
| Pham et al. (2014)(53) | coffee and caffeine | depressive symptoms | NR | Japan | July and November 2009 | cross-sectional study | NR | 567 | Center for Epidemiologic Studies Depression Scale (CES-D) | ≥16 | NR | NR | NR | NR | 20-68 | NR | 57.32% | 157 | NR | NR | NR | NR | 6 | moderate |
| Guo et al. (2014)(54) | sweetened beverages; soft drinks; fruit drinks; coffee | depression disorder | The NIH-AARP Diet and Health study | United States | 1995-1996,2000 | prospective cohort study | NR | 263,923 | NR | reported a diagnosis of depression | NR | NR | NR | NR | 50-71 | NR | 60.66% | 11,311 | NR | NR | 61.1 (5.6) | 48.50% | 9 | high |
| Azagba et al. (2014)(55) | caffeinated energy drink | depressive symptoms | Student Drug Use Survey in the Atlantic Provinces (SDUSAP) | Canada | 2012 | cross-sectional study | stratified multistage clustered sampling | 8,210 | 12 item version of the Centers for Epidemiological Studies Depression Scale (CES-D) | ≥12 | NR | NR | NR | NR | NR | 15.2 | 48.20% | NR | NR | NR | NR | NR | 6 | moderate |
| Lucas et al. (2011)(56) | coffee and caffeine | depression disorder | The Nurses’ Health Study | United States | 1996 baseline, follow until 2006 | prospective cohort study | random sampling | 50,739 | NR | reporting both a new physician-diagnosed depression and beginning regular antidepressant use | NR | NR | NR | NR | 50-75 | 63 | 0 | 2,607 | NR | NR | NR | 0 | 9 | high |
| Shi et al. (2010)(57) | soft drinks | depression disorder, anxiety disorder, stress | South Australian Monitoring and Surveillance System (SAMSS) | Australia | from March 2008 to December 2008 | cross-sectional study | random sampling | 4,741 | NR | doctor-diagnose | NR | doctor-diagnose | NR | doctor-diagnose | ≥16 | 46.7 | 48.74% | NR | NR | NR | NR | NR | 6 | moderate |
| Ruusunen et al. (2010)(58) | coffee and caffeine | major depression disorder | Kuopio Ischaemic Heart Disease Risk Factor Study (KIHD) | Finland | baseline: 1984-1989, followed until the end of 2006 | cohort study | NR | 2,232 | NR | doctor-diagnose | NR | NR | NR | NR | 42;48;54;60 | 53.0 (5.2) | 100% | 49 | NR | NR | 51.6 (5.8) | 100% | 8 | high |
| Hintikka et al. (2005)(59) | coffee | depressive symptoms | Kuopio Depression (KU- DEP) Study | Finland | in April 1998 | cross-sectional study | multistage stratified sampling | 2,011 | Beck Depression Inventory (BDI) | ≥15 | NR | NR | NR | NR | 25-64 | NR | 44.26% | 235 | NR | NR | NR | NR | 6 | moderate |

| Table S4. Quality assessment of included cross-sectional studies | | | | | | | | | | | | |
| --- | --- | --- | --- | --- | --- | --- | --- | --- | --- | --- | --- | --- |
| Study | Item1 | Item2 | Item3 | Item4 | Item5 | Item6 | Item7 | Item8 | Item9 | Item10 | Item11 | Total score |
| Yang et al.-a (2024) | 1 | 1 | 0 | 0 | 0 | 0 | 1 | 1 | 1 | 1 | 0 | 6 |
| Yang et al.-b (2024) | 1 | 1 | 1 | 1 | 0 | 0 | 0 | 1 | 0 | 1 | 0 | 6 |
| Tang et al. (2024) | 1 | 1 | 1 | 1 | 0 | 0 | 0 | 1 | 0 | 0 | 0 | 5 |
| Qiu et al. (2024) | 1 | 1 | 0 | 0 | 0 | 0 | 1 | 1 | 1 | 1 | 0 | 6 |
| Li et al. (2024) | 1 | 1 | 1 | 0 | 0 | 0 | 1 | 1 | 0 | 1 | 0 | 6 |
| Ikram et al. (2024) | 1 | 1 | 0 | 0 | 0 | 0 | 1 | 1 | 0 | 0 | 0 | 4 |
| Cho et al. (2024) | 1 | 1 | 1 | 0 | 0 | 0 | 0 | 1 | 0 | 0 | 0 | 4 |
| Bahrami et al. (2024) | 1 | 1 | 1 | 1 | 0 | 0 | 1 | 1 | 0 | 0 | 0 | 6 |
| Zhang et al. (2023) | 1 | 1 | 1 | 0 | 0 | 0 | 0 | 1 | 0 | 0 | 0 | 4 |
| Yin et al. (2023) | 1 | 1 | 1 | 0 | 0 | 1 | 1 | 1 | 1 | 1 | 0 | 8 |
| Sahril et al. (2023) | 1 | 1 | 1 | 1 | 0 | 0 | 1 | 1 | 0 | 0 | 0 | 6 |
| Luo et al. (2023) | 1 | 1 | 1 | 0 | 0 | 0 | 1 | 0 | 0 | 0 | 0 | 4 |
| Wu et al. (2022) | 1 | 0 | 0 | 0 | 0 | 0 | 0 | 1 | 0 | 0 | 0 | 2 |
| Ra (2022) | 1 | 1 | 1 | 1 | 0 | 0 | 1 | 1 | 0 | 1 | 0 | 7 |
| Nouri-Majd et al. (2022) | 1 | 1 | 1 | 1 | 0 | 1 | 1 | 1 | 0 | 0 | 0 | 7 |
| Long et al. (2022) | 1 | 1 | 1 | 1 | 0 | 0 | 1 | 1 | 0 | 0 | 0 | 6 |
| Liu et al.-a (2022) | 1 | 1 | 1 | 1 | 0 | 0 | 1 | 1 | 0 | 0 | 0 | 6 |
| Liu et al.-b (2022) | 1 | 0 | 1 | 0 | 0 | 0 | 0 | 1 | 0 | 0 | 0 | 3 |
| Kim et al. (2022) | 1 | 1 | 1 | 0 | 0 | 0 | 1 | 1 | 0 | 0 | 0 | 5 |
| Fond et al. (2022) | 1 | 1 | 1 | 1 | 0 | 0 | 1 | 1 | 0 | 0 | 0 | 6 |
| Canas-Simião et al. (2022) | 1 | 0 | 1 | 1 | 0 | 0 | 0 | 1 | 0 | 0 | 0 | 4 |
| Bi et al. (2022) | 1 | 1 | 1 | 1 | 0 | 0 | 1 | 1 | 1 | 1 | 0 | 8 |
| Bharati et al. (2022) | 1 | 1 | 1 | 1 | 0 | 1 | 1 | 1 | 0 | 1 | 0 | 8 |
| Bakhriansyah et al. (2022) | 1 | 1 | 1 | 1 | 0 | 0 | 1 | 1 | 0 | 0 | 0 | 6 |
| Yim et al. (2021) | 1 | 1 | 1 | 0 | 0 | 0 | 0 | 1 | 0 | 1 | 0 | 5 |
| Michels et al. (2021) | 1 | 1 | 1 | 1 | 0 | 0 | 1 | 1 | 0 | 1 | 0 | 7 |
| Kleppang et al. (2021) | 1 | 1 | 1 | 0 | 0 | 0 | 0 | 1 | 0 | 1 | 0 | 5 |
| Kim et al. (2021) | 1 | 1 | 1 | 1 | 0 | 0 | 1 | 1 | 0 | 0 | 0 | 6 |
| Collins et al. (2021) | 1 | 1 | 1 | 1 | 0 | 0 | 1 | 1 | 0 | 1 | 0 | 7 |
| Sangsefidi et al. (2020) | 1 | 0 | 1 | 1 | 0 | 0 | 0 | 1 | 0 | 0 | 0 | 4 |
| Miller et al. (2020) | 1 | 1 | 1 | 1 | 0 | 0 | 1 | 1 | 0 | 1 | 0 | 7 |
| Kimura et al. (2020) | 1 | 1 | 1 | 0 | 0 | 0 | 1 | 1 | 0 | 1 | 0 | 6 |
| Khan et al. (2020) | 1 | 1 | 1 | 1 | 0 | 0 | 0 | 1 | 0 | 1 | 0 | 6 |
| Sousa et al. (2019) | 1 | 1 | 0 | 1 | 0 | 0 | 0 | 1 | 0 | 0 | 0 | 4 |
| Pengpid et al.-a (2019) | 1 | 0 | 1 | 0 | 0 | 0 | 0 | 1 | 0 | 0 | 0 | 3 |
| Pengpid et al.-b (2019) | 1 | 0 | 0 | 0 | 0 | 0 | 0 | 1 | 0 | 0 | 0 | 2 |
| Park et al. (2018) | 1 | 0 | 0 | 1 | 0 | 0 | 0 | 1 | 0 | 0 | 0 | 3 |
| Masana et al. (2018) | 1 | 1 | 1 | 0 | 0 | 0 | 0 | 1 | 0 | 0 | 0 | 4 |
| Kim et al. (2018) | 1 | 1 | 1 | 0 | 0 | 0 | 1 | 1 | 0 | 1 | 0 | 6 |
| Chan et al. (2018) | 1 | 1 | 1 | 1 | 0 | 0 | 0 | 1 | 0 | 0 | 0 | 5 |
| Yu et al. (2017) | 1 | 1 | 1 | 0 | 0 | 0 | 0 | 1 | 1 | 1 | 0 | 6 |
| Kim et al. (2017) | 1 | 1 | 1 | 0 | 0 | 0 | 1 | 1 | 0 | 1 | 0 | 6 |
| Yu et al. (2015) | 1 | 1 | 0 | 0 | 0 | 1 | 1 | 1 | 0 | 0 | 0 | 5 |
| Park et al. (2015) | 1 | 1 | 1 | 0 | 0 | 0 | 1 | 1 | 0 | 0 | 0 | 5 |
| Zahedi et al. (2014) | 1 | 1 | 1 | 1 | 0 | 0 | 0 | 1 | 0 | 0 | 0 | 5 |
| Trapp et al. (2014) | 1 | 1 | 1 | 1 | 1 | 0 | 1 | 1 | 0 | 1 | 1 | 9 |
| Pham et al. (2014) | 1 | 1 | 1 | 0 | 0 | 0 | 1 | 1 | 0 | 1 | 0 | 6 |
| Azagba et al. (2014) | 1 | 1 | 1 | 1 | 0 | 0 | 1 | 1 | 0 | 0 | 0 | 6 |
| Shi et al. (2010) | 1 | 1 | 1 | 1 | 0 | 0 | 0 | 1 | 0 | 1 | 0 | 6 |
| Hintikka et al. (2005) | 1 | 1 | 1 | 1 | 0 | 0 | 1 | 1 | 0 | 0 | 0 | 6 |
| Note: Item 1: Was the source of information (survey, record review) clearly defined? Item 2: Were inclusion and exclusion criteria for exposed and unexposed subjects (cases and controls) explicitly listed or referenced in previous publications? Item 3: Was the time period used for identifying patients clearly indicated? Item 4: If the study was not population-based, were subjects enrolled consecutively? Item 5: Were evaluators of subjective components masked (blinded) to other aspects of the participants’ status? Item 6: Were any assessments undertaken for quality assurance purposes (e.g., test/retest of primary outcome measurements) described? Item 7: Were any patient exclusions from the analysis clearly explained? Item 8: Was the assessment and/or control of confounding described? Item 9: If applicable, was the method for handling missing data explained? Item 10: Were patient response rates and the completeness of data collection summarized? Item 11: Was the expected follow-up clarified, and was the percentage of patients with incomplete data or loss to follow-up reported? | | | | | | | | | | | | |

| Table S5. Quality assessment of included cohort studies | | | | | | | | | |
| --- | --- | --- | --- | --- | --- | --- | --- | --- | --- |
| Study | Representativeness of the exposure group | Selection of the non-exposure group | Ascertainment of exposure | Outcome not present at start | Comparability on the basis of the analysis | Assessment of outcome | Long enough follow-up for outcome (≥2 years) | Completeness of follow-up | Total score |
| Liu et al. (2024) |  | ☆ |  | ☆ | ☆☆ |  |  | ☆ | 5 |
| Chen et al. (2024) | ☆ | ☆ |  | ☆ | ☆☆ | ☆ | ☆ | ☆ | 8 |
| Min et al. (2023) | ☆ | ☆ |  | ☆ | ☆☆ |  | ☆ | ☆ | 7 |
| Kashino et al. (2021) |  | ☆ |  | ☆ | ☆☆ |  | ☆ | ☆ | 6 |
| Elstgeest et al. (2019) | ☆ | ☆ |  | ☆ | ☆☆ |  | ☆ | ☆ | 7 |
| Sanchez-Villegas et al. (2018) |  | ☆ |  | ☆ | ☆☆ | ☆ | ☆ | ☆ | 7 |
| Navarro et al. (2018) |  | ☆ |  | ☆ | ☆☆ | ☆ | ☆ | ☆ | 7 |
| Guo et al. (2014) | ☆ | ☆ | ☆ | ☆ | ☆☆ | ☆ | ☆ | ☆ | 9 |
| Lucas et al. (2011) | ☆ | ☆ | ☆ | ☆ | ☆☆ | ☆ | ☆ | ☆ | 9 |
| Ruusunen et al. (2010) | ☆ | ☆ |  | ☆ | ☆☆ | ☆ | ☆ | ☆ | 8 |

 Fig. S1. Global distribution of odds ratios for non-alcoholic beverage consumption and mental health (depression, anxiety, or stress)

Fig. S2. Forest plot of the association between non-alcoholic beverage consumption and depression risk

Fig. S3. Forest plot of gender-specific associations of non-alcoholic beverages with depression

Fig. S4. Sensitivity analysis of the association between non-alcoholic beverage consumption and depression risk

Fig. S5. Forest plot of the association between non-alcoholic beverage consumption and anxiety risk

Fig. S6. Forest plot of gender-specific associations of non-alcoholic beverages with anxiety

Fig. S7. Sensitivity analysis of the association between non-alcoholic beverage consumption and anxiety risk

Fig. S8. Forest plot of the association between non-alcoholic beverage consumption and stress risk

Fig. S9. Forest plot of gender-specific associations of non-alcoholic beverages with stress

Fig. S10. Sensitivity analysis of the association between non-alcoholic beverage consumption and stress risk

Fig. S11. Meta-regression bubble plots of male proportion and quality score on the association between non-alcoholic beverages and depression, anxiety, stress

**Included references of meta-analysis**

1.	Yang Y, Liu J, Dai D. Associations between sugar-sweetened beverages consumption, duration of physical exercise, and depressive symptoms among Tibetan university students at high altitude. Front Psychol. 2024;15:1439451.

2.	Yang J, Ning M, Tian Y, Chen Z, Liu Y, Yu Q, et al. Association between bubble tea consumption and mental health among adults in mainland China: A national cross-sectional analysis of cohort study. J Affect Disord. 2024 Oct 22;369:1115–21.

3.	Tang WK, Lee JCY. Association of Fast-Food Intake with Depressive and Anxiety Symptoms among Young Adults: A Pilot Study. Nutrients. 2024 Sep 30;16(19).

4.	Qiu Q, Chai G, Xie S, Wu T. Association of sugar-sweetened beverage consumption and sleep quality with anxiety symptoms: a cross-sectional study of Tibetan college students at high altitude. Front Psychol. 2024;15:1383042.

5.	Liu Q, Peng S, Jiang W, He Y, Lu C, Wang W, et al. Associations of black and sugar-sweetened coffee consumption with depressive symptoms: A longitudinal study of Chinese adolescents. J Affect Disord. 2024 Oct 3;369:338–44.

6.	Li L, Yang P, Duan Y, Xie J, Liu M, Zhou Y, et al. Association between dietary diversity, sedentary time outside of work and depressive symptoms among knowledge workers: a multi-center cross-sectional study. BMC Public Health. 2024 Jan 2;24(1):53.

7.	Ikram M, Shahbaz H, Ahmed W, Khan Z, Tahir S, Akram K. Higher Intakes of Soft Drinks Are Associated With Anxiety, Depression, and Bone Mineral Density Among University Students. Top Clin Nutr. 2024 Jan;39(1):33–45.

8.	Cho J, Kim S, Shin H, Kim H, Park E. The Association between High-Caffeine Drink Consumption and Anxiety in Korean Adolescents. NUTRIENTS. 2024 Mar;16(6).

9.	Chen Y, Yang H, Sheng B, Zhou L, Li D, Zhang M, et al. Consumption of sugary beverages, genetic predisposition and the risk of depression: a prospective cohort study. Gen Psychiatry. 2024;37(4):e101446.

10.	Bahrami G, Mohammadifard N, Haghighatdoost F, Emamjomeh A, Najafi F, Farshidi H, et al. The association between soft drinks consumption and risk of mental disorders among Iranian adults: The LIPOKAP study. J Affect Disord. 2024 Oct 15;363:8–14.

11.	Zhang Y, Tao S, Qu Y, Mou X, Gan H, Zhou P, et al. The correlation between lifestyle health behaviors, coping style, and mental health during the COVID-19 pandemic among college students: Two rounds of a web-based study. Front Public Health. 2022;10:1031560.

12.	Yin J, Ding Y, Xu F, Zhao L, Gong R, Yang J, et al. Does the timing of intake matter? Association between caffeine intake and depression: Evidence from the National Health and Nutrition Examination Survey. J Affect Disord. 2023 Nov 1;340:362–8.

13.	Sahril N, Adnan M, Khalil M, Chan Y, Ratnam K, Lai W, et al. Association of dietary behaviour and depression among adolescents in Malaysia: a cross-sectional study. J Health Popul Nutr. 2023 Nov 28;42(1).

14.	Min J, Cao Z, Cui L, Li F, Lu Z, Hou Y, et al. The association between coffee consumption and risk of incident depression and anxiety: Exploring the benefits of moderate intake. Psychiatry Res. 2023 Aug;326:115307.

15.	Luo Y, Yang P, Wan Z, Kang Y, Dong X, Li Y, et al. Dietary diversity, physical activity and depressive symptoms among middle-aged women: A cross-sectional study of 48,637 women in China. J Affect Disord. 2023 Jan 15;321:147–52.

16.	Wu Y, Lu Y, Xie G. Bubble tea consumption and its association with mental health symptoms: An observational cross-sectional study on Chinese young adults. J Affect Disord. 2022 Feb 15;299:620–7.

17.	Ra JS. Consumption of sugar-sweetened beverages and fast foods deteriorates adolescents’ mental health. Front Nutr. 2022;9:1058190.

18.	Nouri-Majd S, Salari-Moghaddam A, Keshteli A, Afshar H, Esmaillzadeh A, Adibi P. Coffee and caffeine intake in relation to symptoms of psychological disorders among adults. PUBLIC Health Nutr. 2022 Dec;25(12):3509–19.

19.	Long Z, Huang L, Lyu J, Xia Y, Chen Y, Li R, et al. The associations of perceived stress with food preferences and activity preferences: A cross-sectional study among Chinese adults. J Affect Disord. 2022 Jan 15;297:430–6.

20.	Liu J, Chen T, Chen M, Ma Y, Ma T, Gao D, et al. Sugar-Sweetened Beverages and Depressive and Social Anxiety Symptoms Among Children and Adolescents Aged 7-17 Years, Stratified by Body Composition. Front Nutr. 2022;9:888671.

21.	Liu BP, Jia CX, Li SX. Soft drink consumption and depressive symptoms among the adolescents of United States: The mediating role of aggressive behaviors. J Affect Disord. 2022 Oct 15;315:27–34.

22.	Kim J, Hong C, Lee G. Association between sugar-sweetened beverage consumption and depression and suicidal ideation among Korean adults: a cross-sectional study from the 2014 and 2016 Korean National Health and Nutrition Examination Survey (KNHANES). Nutr Res Pract. 2022 Apr;16(2):194–204.

23.	Fond G, Boulangeat C, Messiaen M, Duba A, Boucekine M, Auquier P, et al. Anxiety and depression in young physicians: Prevalence and associated factors. The MESSIAEN national study. L’Encephale. 2022 Feb;48(1):26–30.

24.	Canas-Simiao H, Reis C, Carreiras D, Espada-Santos P, Paiva T. Health-Related Behaviors and Perceived Addictions Predictors of Depression During the COVID Lockdown. J Nerv Ment Dis. 2022 Aug;210(8):613–21.

25.	Bi C, Lin H, Zhang J, Zhao Z. Association between Sleep Quality and Depression Symptoms in Chinese College Students during the COVID-19 Lockdown Period. Child-BASEL. 2022 Aug;9(8).

26.	Bharati DR, Kumari S, Prasad N, Choudhary SK, Kumar S, Pal R. Correlates of depression among school going adolescents in the urban area of Patna in eastern India. J Fam Med Prim Care. 2022 May;11(5):1702–9.

27.	Bakhriansyah M, Sulaiman S, Fauzia R. The effect of Camellia sinensis tea on a decreased risk of anxiety for medical students at Universitas Lambung Mangkurat Indonesia. Clin Epidemiol Glob Health. 2022 Sep;17.

28.	Yim HR, Yun HJ, Lee JH. An Investigation on Korean Adolescents’ Dietary Consumption: Focused on Sociodemographic Characteristics, Physical Health, and Mental Health. Int J Environ Res Public Health. 2021 Sep 16;18(18).

29.	Michels N. Poor Mental Health Is Related to Excess Weight via Lifestyle: A Cross-Sectional Gender- and Age-Dependent Mediation Analysis. Nutrients. 2021 Jan 28;13(2).

30.	Kleppang A, Haugland S, Bakken A, Stea T. Lifestyle habits and depressive symptoms in Norwegian adolescents: a national cross-sectional study. BMC PUBLIC Health. 2021 Apr 28;21(1).

31.	Kim JM, Lee E. Association between Soft-Drink Intake and Obesity, Depression, and Subjective Health Status of Male and Female Adults. Int J Environ Res Public Health. 2021 Oct 3;18(19).

32.	Kashino I, Kochi T, Imamura F, Eguchi M, Kuwahara K, Nanri A, et al. Prospective association of soft drink consumption with depressive symptoms. Nutr Burbank Los Angel Cty Calif. 2021 Jan;81:110860.

33.	Collins S, Lotfalian M, Marx W, Lane M, Allender S, Jacka F, et al. Associations between indicators of diet quality and psychological distress, depression and anxiety in emerging adults: Results from a nationally representative observational sample. Ment Health Prev [Internet]. 2021 Dec;24. Available from: https://search.ebscohost.com/login.aspx?direct=true&db=psyh&AN=2022-09762-001&lang=zh-cn&site=ehost-live

34.	Sangsefidi Z, Lorzadeh E, Hosseinzadeh M, Mirzaei M. Dietary habits and psychological disorders in a large sample of Iranian adults: a population-based study. Ann Gen PSYCHIATRY. 2020 Feb 25;19(1).

35.	Miller C, Ettridge K, Wakefield M, Pettigrew S, Coveney J, Roder D, et al. Consumption of Sugar-Sweetened Beverages, Juice, Artificially-Sweetened Soda and Bottled Water: An Australian Population Study. NUTRIENTS. 2020 Mar;12(3).

36.	Kimura Y, Suga H, Kobayashi S, Sasaki S. Intake of Coffee Associated With Decreased Depressive Symptoms Among Elderly Japanese Women: A Multi-Center Cross-Sectional Study. J Epidemiol. 2020 Aug 5;30(8):338–44.

37.	Khan A, Ahmed R, Burton N. Prevalence and correlates of depressive symptoms in secondary school children in Dhaka city, Bangladesh. Ethn Health. 2020 Jan 2;25(1):34–46.

38.	Sousa KT de, Marques ES, Levy RB, Azeredo CM. Food consumption and depression among Brazilian adults: results from the Brazilian National Health Survey, 2013. Cad Saude Publica. 2019;36(1):e00245818.

39.	Pengpid S, Peltzer K. High Carbonated Soft Drink Intake is Associated with Health Risk Behavior and Poor Mental Health among School-Going Adolescents in Six Southeast Asian Countries. Int J Environ Res Public Health. 2019 Dec 23;17(1).

40.	Pengpid S, Peltzer K. High carbonated soft drink consumption is associated with externalizing but not internalizing behaviours among university students in five ASEAN states. Psychol Res Behav Manag. 2019;12:585–92.

41.	Elstgeest LEM, Visser M, Penninx BWJH, Colpo M, Bandinelli S, Brouwer IA. Bidirectional associations between food groups and depressive symptoms: longitudinal findings from the Invecchiare in Chianti (InCHIANTI) study. Br J Nutr. 2019 Feb;121(4):439–50.

42.	Sanchez-Villegas A, Zazpe I, Santiago S, Perez-Cornago A, Martinez-Gonzalez MA, Lahortiga-Ramos F. Added sugars and sugar-sweetened beverage consumption, dietary carbohydrate index and depression risk in the Seguimiento Universidad de Navarra (SUN) Project. Br J Nutr. 2018 Jan;119(2):211–21.

43.	Park S, Rim SJ, Lee JH. Associations between dietary behaviours and perceived physical and mental health status among Korean adolescents. Nutr Diet J Dietit Assoc Aust. 2018 Nov;75(5):488–93.

44.	Navarro AM, Abasheva D, Martínez-González MÁ, Ruiz-Estigarribia L, Martín-Calvo N, Sánchez-Villegas A, et al. Coffee Consumption and the Risk of Depression in a Middle-Aged Cohort: The SUN Project. Nutrients. 2018 Sep 19;10(9).

45.	Masana M, Haro J, Mariolis A, Piscopo S, Valacchi G, Bountziouka V, et al. Mediterranean diet and depression among older individuals: The multinational MEDIS study. Exp Gerontol. 2018 Sep;110:67–72.

46.	Kim J, Kim J. Green Tea, Coffee, and Caffeine Consumption Are Inversely Associated with Self-Report Lifetime Depression in the Korean Population. Nutrients. 2018 Sep 1;10(9).

47.	Chan SP, Yong PZ, Sun Y, Mahendran R, Wong JCM, Qiu C, et al. Associations of Long-Term Tea Consumption with Depressive and Anxiety Symptoms in Community-Living Elderly: Findings from the Diet and Healthy Aging Study. J Prev Alzheimers Dis. 2018;5(1):21–5.

48.	Yu ZM, Parker L, Dummer TJB. Associations of Coffee, Diet Drinks, and Non-Nutritive Sweetener Use with Depression among Populations in Eastern Canada. Sci Rep. 2017 Jul 24;7(1):6255.

49.	Kim S, Sim S, Choi H. High stress, lack of sleep, low school performance, and suicide attempts are associated with high energy drink intake in adolescents. PLOS ONE. 2017 Nov 14;12(11).

50.	Yu B, He H, Zhang Q, Wu H, Du H, Liu L, et al. Soft drink consumption is associated with depressive symptoms among adults in China. J Affect Disord. 2015 Feb 1;172:422–7.

51.	Zahedi H, Kelishadi R, Heshmat R, Motlagh ME, Ranjbar SH, Ardalan G, et al. Association between junk food consumption and mental health in a national sample of Iranian children and adolescents: the CASPIAN-IV study. Nutr Burbank Los Angel Cty Calif. 2014 Dec;30(11–12):1391–7.

52.	Trapp G, Allen K, O’Sullivan T, Robinson M, Jacoby P, Oddy W. Energy drink consumption is associated with anxiety in Australian young adult males. Depress ANXIETY. 2014 May;31(5):420–8.

53.	Pham N, Nanri A, Kurotani K, Kuwahara K, Kume A, Sato M, et al. Green tea and coffee consumption is inversely associated with depressive symptoms in a Japanese working population. PUBLIC Health Nutr. 2014 Mar;17(3):625–33.

54.	Guo X, Park Y, Freedman ND, Sinha R, Hollenbeck AR, Blair A, et al. Sweetened beverages, coffee, and tea and depression risk among older US adults. PloS One. 2014;9(4):e94715.

55.	Azagba S, Langille D, Asbridge M. An emerging adolescent health risk: Caffeinated energy drink consumption patterns among high school students. Prev Med. 2014 May;62:54–9.

56.	Lucas M, Mirzaei F, Pan A, Okereke OI, Willett WC, O’Reilly ÉJ, et al. Coffee, caffeine, and risk of depression among women. Arch Intern Med. 2011 Sep 26;171(17):1571–8.

57.	Shi Z, Taylor AW, Wittert G, Goldney R, Gill TK. Soft drink consumption and mental health problems among adults in Australia. Public Health Nutr. 2010 Jul;13(7):1073–9.

58.	Ruusunen A, Lehto SM, Tolmunen T, Mursu J, Kaplan GA, Voutilainen S. Coffee, tea and caffeine intake and the risk of severe depression in middle-aged Finnish men: the Kuopio Ischaemic Heart Disease Risk Factor Study. Public Health Nutr. 2010 Aug;13(8):1215–20.

59.	Hintikka J, Tolmunen T, Honkalampi K, Haatainen K, Koivumaa-Honkanen H, Tanskanen A, et al. Daily tea drinking is associated with a low level of depressive symptoms in the Finnish general population. Eur J Epidemiol. 2005;20(4):359–63.
